# Gut Microbiota Metabolism and Interaction with Food Components

**DOI:** 10.3390/ijms21103688

**Published:** 2020-05-23

**Authors:** Pamela Vernocchi, Federica Del Chierico, Lorenza Putignani

**Affiliations:** 1 Unit of Human Microbiome, Bambino Gesù Children’s Hospital, IRCCS, Viale San Paolo 15, 00146 Rome, Italy; federica.delchierico@opbg.net; 2 Unit of Parasitology and Unit of Human Microbiome, Bambino Gesù Children’s Hospital, IRCCS, Piazza Sant’ Onofrio 4, 00165 Rome, Italy; lorenza.putignani@opbg.net

**Keywords:** microbiota metabolism, diet, metabolome, microbiome therapeutics

## Abstract

The human gut contains trillions of microbes that play a central role in host biology, including the provision of key nutrients from the diet. Food is a major source of precursors for metabolite production; in fact, diet modulates the gut microbiota (GM) as the nutrients, derived from dietary intake, reach the GM, affecting both the ecosystem and microbial metabolic profile. GM metabolic ability has an impact on human nutritional status from childhood. However, there is a wide variability of dietary patterns that exist among individuals. The study of interactions with the host via GM metabolic pathways is an interesting field of research in medicine, as microbiota members produce myriads of molecules with many bioactive properties. Indeed, much evidence has demonstrated the importance of metabolites produced by the bacterial metabolism from foods at the gut level that dynamically participate in various biochemical mechanisms of a cell as a reaction to environmental stimuli. Hence, the GM modulate homeostasis at the gut level, and the alteration in their composition can concur in disease onset or progression, including immunological, inflammatory, and metabolic disorders, as well as cancer. Understanding the gut microbe–nutrient interactions will increase our knowledge of how diet affects host health and disease, thus enabling personalized therapeutics and nutrition.

## 1. Introduction

The human gastrointestinal (GI) tract contains many mutualistic microbes (over 10 trillion microbial cells), providing numerous specialized metabolites, small and other bioactive molecules that trigger immune and host metabolic pathways. Hence, the gut microbiota (GM) is also called a “metabolic organ” with a metabolic potential, comparable to the same as that which the liver has [1].

The innovation in metabolomics and metagenomics disciplines has allowed the discovery of many microbe-derived small molecules, as well as the genes linked to their production [2]. Hence, the symbiotic relation between GM and host produces a myriad of metabolic signatures, and the technological advances in GM metabolomics are progressively decoding the host–microbes metabolic interaction [3].

GM provides primary metabolites, converting these small molecules into secondary metabolites named “specialized metabolites” [4,5].

Several specialized metabolites show connections with biosynthetic pathways that are unique to an organism or to their class, and by metagenome and metatranscriptome sequencing, combination is possible in order to assign taxonomy and functions [6]. Moreover, the high resolution of mass spectrometry coupled with molecular networking analysis, corroborated with computational approaches, will allow for the understanding of host–microbiome crosstalk mediated by the associated specialized metabolites [5].

Microbial ecosystems may rapidly shift their functionality in response to dietary changes, enhancing human dietary elasticity [7]. Among different environmental variables, short-term dietary interventions coupled with long-term dietary patterns regulate the GM physiology [8].

The processes related to the metabolism of nutrients and xenobiotics affect the following mechanisms: (i) chemical crosstalk between microbial and human metabolic compounds [9,10], (ii) modulation of immune system [11], (iii) protection from pathogens [12], (iv) enteric nervous system regulation [13], (v) colorectal cancer resistance [14,15,16], (vi) neurological behavior [17], and (vii) reduction of lipid levels in serum and cholesterol balancing [18].

In particular, by deepening the concepts, the effect of the environment (nutrition, social, behavioral, geography) on host genetics and the following GM adaptation, triggers the molecular mechanisms of communication between the microbiome and the host, which is called crosstalk.

Crosstalk could provide an explanation of the development of these diseases, which will help to highlight potential targets and biomarkers able to modulate the abundance of metabolites and relevant functions, as well as operational taxonomic units (OTUs) for health, for further investigations on potential new metabolic functions of the GM [19].

Recently, the interest has grown in terms of understanding of how commensal bacteria metabolites derived from interaction with nutrients could regulate the host immune system. Commensal bacteria are important digestion regulators, and the intestinal content is a mix of microbes that are important for the processing and absorption of several nutrients and metabolites, including bile acids, lipids, amino acids (AAs), vitamins, and short-chain fatty acids (SCFAs) [11]. These nutrients and metabolites, which derive from commensal bacteria, are directly linked to diet and digestion [20], and may modulate immune cells through direct and indirect mechanisms in the context of health and disease.

SCFAs, particularly propionate and butyrate, could downregulate the gene expression of pathogenicity island 1 in *Salmonella typhimurium* required for intestinal epithelial cell invasion [21]. Additionally, the microbial-synthetized molecules and host-derived molecules can be metabolized by commensals, resulting, for example, in the production of primary bile acids following conversion into secondary bile acids, which play a crucial role in defense against pathogens such as the suppression of *Clostridium difficile* growth [22].

Special attention can also be given to the enteric nervous system (ENS), which controls major GI functions independently of the central nervous system. Recent evidence has shown that butyrate, a molecule belonging to SCFAs, can modulate neuronal functions by gene expression of neuromodulators, as well as GI motility. In particular, butyrate increases the proportion of choline acetyltransferase by the Src-kinase signaling pathway and the acetylation of histone H3K9 in enteric neuron, as well as the motility of colon by the activation of cholinergic pathways [13].

Moreover, in cancer, especially in human colon adenocarcinoma cell line (HT-29), acetate and propionate trigger apoptosis at pH 7.5 and necrosis at pH 5.5. These processes are probably induced by mitochondrial depolarization, inner membrane permeabilization, drastic depletion in adenosine triphosphate (ATP) levels, and reactive oxygen species (ROS) accumulation in HT-29 cells [15].

There are several studies on the effect of butyrate on mitochondrial activity, which has also been studied in lymphoblastoid cell lines (LCL) derived from children with autism spectrum disorder (ASD). In this study, authors demonstrated that butyrate in particular has a positive effect on mitochondrial function in LCLs in the context of physiological stress and/or mitochondrial dysfunction, and can help rescue energy metabolism during disease states, having a potential role in host physiology and behavior in ASD [23].

Moreover, non-digestible/fermentable nutrients could also modulate GM activity, determining cholesterol- or triglyceride-lowering effects. In particular, certain bacteria with probiotic characteristics and prebiotics enhanced bile acid deconjugation and subsequent increasing of fecal bile acid excretion that is involved in cholesterol reduction [24].

The production of a high concentration of propionate in rats, through microbial fermentation of resistant starch or fructans, has been identified as a mechanism to explain the reduction in serum and hepatic cholesterol [18]. Moreover, the level of acetate/propionate ratio that reaches the liver by the portal vein is a potential intermediate that could be used to make predictions on the potential lipid-lowering properties belonging to prebiotics and other fermentable carbohydrates [18]. In addition, Saltzman et al. [25,26] may also have demonstrated the mechanisms by which other non-digestible, lipid-lowering carbohydrates produce their effects in humans, in combination with other nutritional factors such as a vegetarian diet or a diet with a high content of cereal fiber, vegetables, and fruits.

The goal of this review is to give a vision on potential future treatments that will be directed to ameliorate host health by modulation of the GM as target, focusing principally on the bacterial metabolic potential, as well as the taxonomy aspect, in order to discover therapeutic and/or diagnostic targets, and also to describe the interaction between the microbiome–host and environmental and dietary changes [19].

## 2. The Dietary Impact on Gut Microbiota and Metabolic Composition

Foods may be considered to be potential etiopathogenetic factors of GI-related disorders [27]. Long-term dietary intake has an impact on the composition and activity of microbes residing in the gut [28,29], but it remains unclear how fast and how much is the rate of the reproducibility of the human GM to respond to short-range changes in term of macronutrients [7].

Hence, saccharolytic fermentation takes place mainly in the proximal colon, as most bacteria choose to utilize carbohydrates instead of proteins [30]. On the contrary, proteolytic fermentation occurs in the distal colon, producing branched-chain fatty acids (BCFAs) and potentially detrimental metabolites such as ammonia (from amino acid deamination and urea hydrolysis), indoles, and phenols (from amino acid (AAs) carboxylation). Therefore, the intake of foods composed entirely of animal or plant ingredients alters the microbial community [29,31].

An animal-based diet, made up of meat, eggs, and cheese, determines the increase of bile-tolerant microbes such as *Alistipes*, *Bilophila*, and *Bacteroides*, and the decrease of Firmicutes that metabolize dietary plant polysaccharides such as *Roseburia*, *Eubacterium rectale*, and *Ruminococcus bromii* [7].

A high-fat diet, especially in terms of saturated fatty acids, and chronic low-grade tissue inflammation [32] determines a microbial community imbalance, leading to dysbiosis (i.e., increasing at the phylum level of cluster XI of the genus *Clostridium*) and producing an altered metabolic profile in the colon lumen.

Hence, this dysbiosis associated with metabolites (or increased translocation of normal metabolites in the host) is involved in systemic efflux and also in GI disorders [33].

On the contrary, a plant-based diet, which is rich in grains, legumes, fruits, and vegetables, appears to be beneficial for human health by promoting the development of diverse and stable microbial ecosystems. In particular, digestible carbohydrates and fructose have been highlighted to decrease Clostridia and *Bacteroides* [34]. Hence, non-digestible carbohydrates lead to an increase in lactic acid bacteria (LAB), *Ruminococcus*, *Eubacterium rectale*, and *Roseburia*, while reducing *Clostridium* and *Enterococcus* species [34]. Bifidobacteria are also raised up by digestible and non-digestible carbohydrates [35].

The plant-based diet is associated with carbohydrate fermentation products and lower fermentation of AAs [36]. Hence, a plant based diet, may result in an increased Bacteroidetes/Firmicutes ratio and also in a consequent weight loss with the reduction of the energy level extracted from the diet [37].

Moreover, it is possible to divide individuals in two groups according to diet model; in particular, *Bacteroides* are associated with elevated animal protein diet/saturated fat, and *Prevotella* represents the main group associated with an agrarian-type diet, rich in fruit and vegetables, with a high quantity of carbohydrates and simple sugars, and low levels of saturated fats and animal proteins [38,39,40].

Interestingly, Hjorth and co-workers reported that overweight and obese individuals with a high *Prevotella*/*Bacteorides* ratio had more success in losing fat by eating fiber and whole grains with respect to individuals with a low *Prevotella*/*Bacteorides* ratio [41].

The microbial populations in vegan individuals compared to controls showed reduced levels of *Bacteroides* spp., *Bifidobacterium* spp., *Escherichia coli*, and Enterobacteriaceae [42].

Abundance of plant foods produce the increase of *Bifidobacterium* and *Lactobacillus*, which give anti-inflammatory and anti-pathogenic effects and cardiovascular protection [20]. 

In addition, diet also introduces food microorganisms considered as food ingredients into the distal gut in relation to different type of ingested food [43]; hence, foodborne microbes from both diets, including bacteria, fungi, and viruses, transiently colonize the gut. In particular, some diet-induced changes into bacterial groups are gut-related, including obesity [44] and inflammatory bowel diseases (IBDs), representing chronic disease’s epidemic in the Westernized world [45].

Diet can deeply influence disease progression by altering the intestinal microbial composition. It induces pathobiont expansion, with reference to low-abundant species such as *Bilophila wadsworthia*, a sulfite-reducing anaerobe that triggers inflammation through canonical activation of dendritic cells presenting antigen to naive T cells [46].

## 3. Health Effects Mediated by Food–Microbiota Metabolome

GM is principally related with (i) dietary fiber degradation, (ii) fermentation and anaerobic degradation of proteins and peptides, (iii) glycoconjugates derivation from the host, (iv) bile acid deconjugation and dihydroxylation, (v) vitamins (B and K) and isoprenoids biosynthesis, (vi) cholesterol decrease and AAs and (vii) xenobiotics degradation in order to provide energy through metabolism [3], (viii) development of nervous system, (ix) regulation of appetite, and (x) intestinal and immune system development. In particular, differences in the intake of dietary macro- and micro-nutrients could directly raise the risk of developing chronic diseases.

In addition, consumption of a plant-based versus Western-type dietary pattern results in different gut bacterial communities [29,47] and metabolite production [7], potentially linked to healthy and disease statuses.

Technology innovation and the advent of big data has extended the knowledge on GM, and has fulfilled the description of small molecule identification such as ribosomally post-translationally modified peptides, lipids and glycolipids, AA metabolites, oligosaccharides and polysaccharides, non-ribosomal peptides, terpenoids, and polyketides [48]. We have grouped the dietary patterns into two macro groups: (1) carbohydrates, fibers, and vitamins, and (2) proteins and fats.

### 3.1. Dietary Carbohydrates, Fibers, and Vitamins

Fermentable carbohydrates, such as pectin gums, hemicelluloses, prebiotic ingredients comprising fructose, fructo-oligosaccharides (FOS), and galactose-oligosaccharides (GOS), are different from dietary fiber such as cellulose or wheat bran, which mammalian enzymes are unable to digest [49].

In particular, anaerobic metabolism of non-digestible carbohydrates performed by gut microbes produces SCFAs and gases from different pathways. SCFAs, such as acetate, propionate, and butyrate, mainly in the lumen, are assumed to interact in terms of the production of health benefits, such as antioxidant and anti-cancer activities previously described, avoiding allergic disorders, and also anti-inflammatory effects on the intestinal mucosa [50,51]. In particular, butyrate and propionate can regulate intestinal physiology and immune function, whereas acetate acts as a substrate for lipogenesis and gluconeogenesis [52].

The microbial species, including SCFA producers, play a key role in the degradation of polysaccharides derived from plants [53], cooperating with bacteria specialized in fermentation of FOS and GOS (i.e., *Bifidobacterium* spp.) in order to generate SCFAs and gas, which are also used as carbon and energy sources by other dedicated bacteria (i.e., sulfate-reducing bacteria and methanogens) [54].

*Clostridium* clusters IV and XIVa (i.e., *Eubacterium*, *Roseburia*, *Faecalibacterium*), and *Lactobacillus* spp. and *Bifidobacterium* spp. represent the principal bacteria that play a key role in SCFAs metabolism [16,55,56].

Recently, important activities have been evidenced for these metabolites in the immune function regulation in the periphery, leading to appropriate immune responses, oral tolerance, and inflammation resolution, as well as the modulation of the inflammatory output of adipose tissue [57].

On the other hand, some water-soluble fibers, such as resistant starch from potatoes, seaweeds [58], and undigested oligosaccharides, are metabolized as SCFAs and lactic acid by colonic bacteria, especially in the distal colon, such as *Faecalibacterium prausnitzii*, *Eubacterium rectale*, *Eubacterium hallii*, and *Ruminococcus bromii* [59]. These can constitute a protective factor in colon carcinogenesis [60]. Regarding colorectal cancer resistance, in in vitro experiments conducted on colon cancer cell lines, propionate, valerate, and especially butyrate caused cellular growth arrest, differentiation, and apoptosis by inhibiting histone deacetylases, consequently leading to the hyperacetylation of selective histone proteins such as histone H4. In the same experimental condition, the previously cited SCFAs altered the expression of the cell cycle regulators p21 and CB1, resulting in growth arrest of colon cancer cells [14].

**Figure 1:**
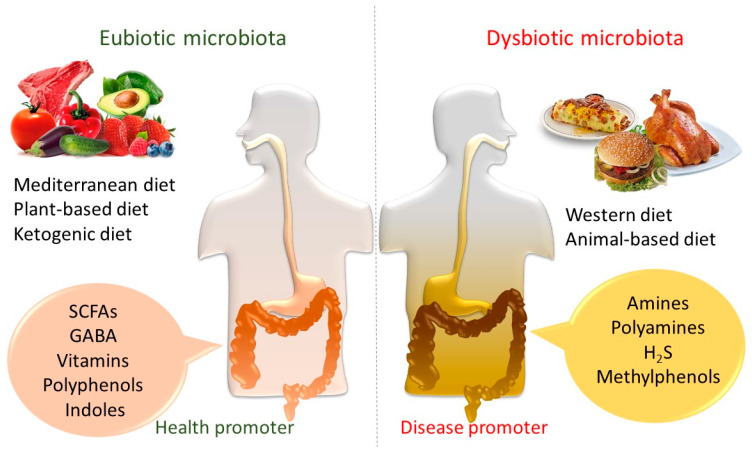
Dietary effect on gut microbiota and health. The gut microbiota takes part in the digestion of food ingredients and in the regulation of host metabolic functions. The nutrient-dependent impact of commensal bacteria to the eubiosis and dysbiosis, thereby the health and disease status, is caused by the production of several microbial metabolites.

Additional functions also include reduction of oxidative stress and reinforcement of the colonic defense barrier [61]. Hence, the principal activities of SCFAs are the following [51]: (1) triggering of Foxp3+ T regulatory (Treg) cells and tolerance; (2) induction of IgA secretion from B cells; (3) “competitive exclusion”, due to high-fiber diet that spreads commensal bacteria and limits the access of pathogenic bacteria to the gut epithelium; (4) promotion of mucus secretion by gut epithelial cells; (5) contribution to the intestinal barrier integrity, in particular by stimulating the formation of the proteins of tight junctions, such as claudin, occludin, and zonulin [62,63], as well as promotion of tissue repair and wound healing; and (6) inhibition of the proinflammatory transcription factor (NF-κB) and decreasing of oxidative stress (Figure 1).

The management, with propionate and butyrate, of human Caco-2/TC7 intestinal epithelial cell line has also determined the reduction of the nine important gene expressions related to cholesterol synthesis, including the key gene HMGCR (3-hydroxy-3-methylglutaryl-coenzyme A reductase).

The Mediterranean diet has a very large fiber content and bioavailability, particularly in terms of insoluble fiber, being more than twofold higher than in a typical Western diet (30 vs. 14 g/day) [64,65].

In a recent randomized clinical trial, Haro et al. [17] took into consideration obese individuals randomized to a Mediterranean diet for 2 years in order to describe the reshaping of the GM, showing an increase in *Bacteroides*; *Prevotella*; and most importantly, in *Roseburia*, *Ruminococcus*, *Parabacteroides distasonis*, and *Faecalibacterium prausnitzii*, which are known for their saccharolytic activity and their ability to digest carbohydrates by producing SCFAs.

When the intestinal SCFA (butyrate) level decreases in patients with IBDs, it is clear that GM diversity decreases by reducing the abundance of Firmicutes and Bacteroidetes and increasing the Proteobacteria level [66].

Furthermore, in patients with recurrent Crohn’s disease, a decreased level of *F. prausnitzii* has been shown, as well as of butyrate, suggesting that the reduction of *F. prausnitzii* may contribute as a marker of a dysbiotic state that predisposes individuals to the IBD [67].

However, in a recent paper it has been reported that *F. prausnitzii* increased in an obese adolescent population and could be considered as a microbial marker in obesity [68]. Indeed, to date, the increasing interest in finding strategies to modulate the abundance of *F. prausnitzii* in the gut are discussed, as well as its usage as a biomarker for diagnostics and prognostics of intestinal diseases [69].

However, in terms of the consumption of high-carbohydrate food (HCF; i.e., glucose, sucrose, pastas, potatoes, white bread), it is possible that it is correlated with obesity, and in late pregnancy, the GM patterns seem to represent a disruptive microbial composition similar to those of adults with type 2 diabetes [70]. HCF consumption is associated with significant changes in the Firmicutes/Bacteroidetes ratio, with a lowering of butyrate-producing bacteria compared to healthy individuals. HCF is usually very deficient of indigestible carbohydrates such as fibers, which provide important physiological benefits such as stimulating incretin production, serving as an energy source for colonic microbes that promote normal bowel movements [71] and the aforementioned production of SCFAs.

Diet provides vitamins, which are absorbed in the small intestine, although the major part of the microbe-mediated vitamin production takes place in the large intestine. Hence, the vitamins produced can be absorbed by the host through specialized carrier-mediated systems, with the exception of cobalamin. Some microbial species such as *Bacteroides* spp., are able to synthesize vitamins *de novo* (vitamin prototrophs) as water-soluble B-vitamins, whereas on the contrary, other species as *Faecalibacterium* spp. that lack biosynthetic pathways require external sources (vitamin auxotrophs) [72,73]. This is why human gut commensals have been known to be important producers of vitamins, which are necessary as essential coenzymes for a wide class of metabolic reactions. Thus, gut microbes can synthesize vitamin K2, as well as water-soluble B-vitamins such as folic acid, niacin, biotin, pantothenic acid, cobalamin, pyridoxine, riboflavin, and thiamine [74,75].

Long-term consumption of plant-based diets with restriction on caloric intake has been associated with abundant and higher microbial phylogenetic differences [76]. Mainly, an increase of lactic acid bacteria (LAB) has been highlighted, such as *Ruminococcus*, *Enterococcus rectale*, and *Roseburia*, as well as increases in *Bifidobacterium* and *Lactobacillus* perhaps due to polyphenols, also abundant in plant foods, which perform anti-inflammatory, anti-pathogenic effects and cardiovascular protection [35].

Furthermore, polyphenols, which are widely distributed in plants, vegetables, and fruits, are also derived from bacterial metabolism of dietary foods at the gut level, and they are converted into derivatives of aromatic SCFAs as phenylacetate or phenylbutyrate.

These metabolites are produced by gut bacteria such as *Bacteroides*, *Clostridium*, *Eubacterium limosum*, and *Eggerthella lenta*, which have a wide range of activities in the prevention and treatment of several diseases such as diabetes, cancer, neuroinflammation, and aging. Indeed, SCFAs and polyphenols, especially phenylbutyrate, inhibit histone deacetylase (HDAC) activity, which is also involved in impaired intestinal epithelial cell function [77,78].

### 3.2. Dietary Proteins and Fats

Numerous studies have shown that high-protein diets in humans make a shift from carbohydrates to protein fermentation through the GM [79], whereas they induce changes, particularly leading to a decrease in hypothetically beneficial microbes that produce butyrate [50].

Animal-based diets are usually energy-full and poor in fiber. The microbes that are not able to digest fibers use proteins, fat, and simple sugars for their growth, and are also more appropriate for harvesting the energy taken in excess, as is the case in the Western diet [80].

On the contrary, diets such as the ketogenic diet (KD) that are high in fat and protein and very low in carbohydrates, are normocaloric diets, in which the glucose in the body becomes deficient for both fat oxidation (oxaloacetate derived from tricarboxylic acid cycle, TCA) and energy, which is required for the central nervous system. Hence, the organism is forced to use fats as a primary fuel, in the form of ketone bodies such as 3-hydroxybutyrate (3HB), acetate, and acetoacetate (AcAc), which are produced in the liver by the ketogenesis process. The KD seems to act as an efficient diet therapy for weight reduction, and is also is used for epilepsy and Glucose Transporter 1 Deficiency Syndrome (GLUT1-DS) [81].

In addition, at the gut level, the KD reduces GM diversity, while increasing the relative abundance of *Akkermansia muciniphila*, *Parabacteroides*, and *Lactobacillus*, which produce SCFAs, and also microbiota-dependent seizure protection has been found to be linked to the increase of gamma-aminobutyric acid (GABA) [82] (Figure 1). Hence, a reduction in pro-inflammatory microbes such as *Desulfovibrio* and *Turicibacter* results in being potentially protected against seizures [82].

Metabolic outcome of protein catabolism by the GM is far more diverse [83], including carbohydrate fermentation that mainly produces SCFAs [84], as well as fermentation of AAs, moreover releasing beneficial SCFAs. This produces, in particular, branched-chain fatty acids (BCFAs), hydrogen sulfide (H_2_S), ammonia, phenolic and indolic compounds, and also amines and polyamines [85]. BCFAs are developed from branched-chain AAs such as valine, leucine, and isoleucine, which give them biomarkers for bacterial proteolysis [86]. The metabolites such as amines, hydrogen sulfide, *p*-cresol, and ammonia, are harmful for the colonic epithelium at excessive concentrations [38,87]. Some of these may play a role in many diseases such as DNA damage, leaky gut, colon cancer, or IBD [54].

On the contrary, molecules such as indolic compounds (precursors of indoxyl sulfate) take part in the maintenance of the epithelial barrier function [12,88].

BCFAs, indoles, and phenols are not generated by human enzymes and therefore they can only derive from bacterial fermentation. The production of these metabolites is often considered as a marker for evaluating the level of protein fermentation in the colon [89].

The exposure to a Western diet poor in “microbiota-reachable carbohydrates” results in the disappearance of specific bacterial lineages, which could harmfully affect the maturation and function of the immune system, as well as causing the growth in the onset risk of a wide range of inflammatory, metabolic, allergic, and autoimmune disorders [90] (Figure 1).

Animal proteins derived from dairy products (caseins) and red meat stimulate the production of genotoxic endogenous *N*-nitroso compounds in the human gut [91], and the levels of fecal *p*-cresol have been significantly increased and correlated with genetic damage [92].

In addition, animal-based diets also contain a higher quantity of choline and L-carnitine, which have been linked to risk of cardiovascular diseases (CVDs) in humans and in mice, due to their conversion into trimethylamine (TMA) by several gut microbes, such as *Candida*, *Campylobacter*, and *Shigella* species, as well as *Ruminococcus gnavus* [93], following the consequent absorption into portal circulation, and the conversion to trimethylamine *N*-oxides (TMAO) in the liver [94].

It has been shown that TMAO decreases the transport of reverse cholesterol and bile acid synthesis, possibly reducing the normal pathway of intestinal cholesterol elimination, thus explaining the relation between bile acid and TMAO regulation emerging as a possible atherosclerosis mediator [95].

TMAO might also be linked to other undesirable conditions, such as obesity, insulin resistance, and gastrointestinal cancers [51,96].

Recently, some studies have detected choline deficiencies in the development of non-alcoholic fatty liver disease (NAFLD), providing evidence of growth of progression to non-alcoholic steatohepatitis (NASH). Even if NAFLD patients remain asymptomatic, 20% progress to NASH can occur, which emerges as increased mortality through cirrhosis, portal hypertension, and hepatocellular carcinoma [97]. Furthermore, by using metagenomics and metabolomics, it was found to be possible to identify a combination of enterotype–metabotype that appeared to contribute to the definition of a signature that changes during the evolution of the disease [98].

On the contrary, vegans and lacto-ovo vegetarians have highlighted the insignificant postprandial value of TMAO in plasma as response to an L-carnitine meal [99]. Diets rich in saturated dietary lipids are associated with growth of white adipose tissue (WAT) and inflammation mediated by GM molecular mechanisms that induces a macrophage increase in WAT and adverse metabolic consequences [100]. The response of gut microbes differs according to the type of dietary fats; for example, in the presence of lard rich in saturated lipids or fish oil, or rich in polyunsaturated lipids. Genera *Bacteroides*, *Turicibacter*, and *Bilophila* were increased in lard-fed mice; on the contrary, Actinobacteria (*Bifidobacterium* and *Adlercreutzia*), LAB (*Lactobacillus* and *Streptococcus*), Verrucomicrobia (*Akkermansia muciniphila*), Alphaproteobacteria, and Deltaproteobacteria were increased in fish oil-fed mice [100]. In particular, *A. muciniphila*, which has been shown to (i) decrease fat mass gain and WAT macrophage infiltration, (ii) increase gut barrier function, (iii) and increase glucose metabolism when given to mice with diet-induced obesity [101].

For other metabolites such as phenols, a current study highlighted that gut bacterial metabolites such as 4-ethylphenylsulfate (4EPS) and indolepyruvate may derived from AA fermentation, potentially triggered ASD in a mouse model [17]. Moreover, several metabolites (such as *p*-cresol and indolyl-3-acryloylglycine), which are similar to 4EPS and indole pyruvate, have been detected in urine as human autism biomarkers [102].

Furthermore, the oral treatment of maternal immune activation (MIA) mouse model with *Bacteroides fragilis* improves gut permeability; modulates the microbial composition and function; and ameliorates deficiencies in communicative, stereotypic, anxiety-like behaviors, suggesting that gut microbiota and host metabolism affects behavior [17].

Finally, the diet-dependent endogenous GM generate metabolites such as low molecular weight compounds such as vitamins (derived directly from dietary or *de novo* synthetized bacteria), polyamines, and SCFAs, as well as diet-independent products such as lipopolysaccharide (LPS) (Gram-negative cell wall component) and peptidoglycan (Gram-positive cell wall component), which interact within the intestinal microenvironment [103] for normal development and behavior [104].

These metabolites also change the epigenome of host cells, and the epigenetic modifications in turn alter the growth and functions of the cell and modulate gene expression [105] during life of each person [106,107].

## 4. Microbiome-Based Therapeutics

The understanding of metabolic capabilities of gut microbial inhabitants in all areas of the human body may be the passport to understanding health- and disease-linked mechanisms.

New knowledge on GM modulation mechanisms can provide potential novel strategies to prevent and treat IBD and extraintestinal inflammatory diseases. Dietary interventions with pre-, pro-, and post-biotics or synbiotics showed an increase in saccharolytic fermentation while concomitantly decreasing proteolytic fermentation [108].

**Figure 2:**
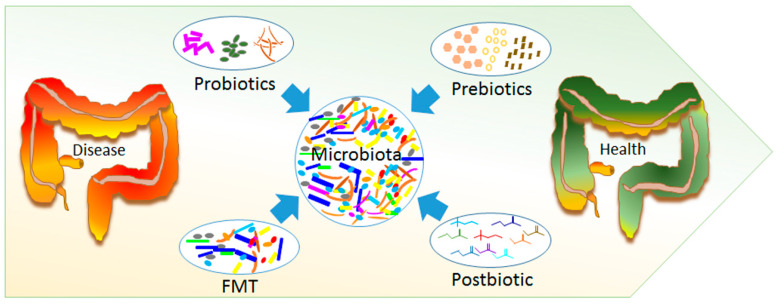
Gut microbiota modulation. Beneficial effect on the intestinal environment through microbial composition modification, by using probiotic, prebiotic, and postbiotic administration and fecal microbiota transplantation (FMT). All these approaches can be optimized by patient-tailored treatments for the better management of the individual’s physiology and pathology.

Thus far, great progress has been reached through microbial analysis tools and therapeutic strategies through dietary intervention with microbes, as well as the usage of fecal microbiota transplantation (FMT) [109] (Figure 2).

Probiotics are defined as live microorganisms that, when administered in adequate amounts, confer a health benefit to the host [110]. Although this definition has been kept constant for 17 years, the transfer of the concept to the public is yet ongoing. In consideration of the wide spectrum of probiotics, many scientists introduce subgrouping, regarding the system targeted or the potential illness. Some probiotic products may have a target disease (i.e., that which is not addressed to the general population but to patients with a specific disease), suitable to be approached by a particular probiotic product [110].

Therefore, the interpretation of probiotic action and their effects on the host is important to modulate the GM and to treat a broad range of human diseases. The actions are commonly related to probiotic use for their anti-microbial effects, mucosal barrier integrity enhancement, and host immunomodulation [109].

The major extensively commercialized probiotics are composed by *Bifidobacterium* and *Lactobacillus* strains, although other microorganisms, such as the yeast *Saccharomyces*, now are also widely employed [111]. However, different probiotic strains are not similarly powerful, and their effects are mediated by host immune response interactions and within a very intricate gut microbial ecosystem. Through targeted or untargeted metagenomics approaches and predicted pathways, researchers may identify the complex relation between microbiota and host, thus providing the development of probiotics for the next generation. Some positive results have been acquired in animal models and human clinical trials by using recombinant LAB, which expresses beneficial molecules to stimulate the suppression of inflammatory systemic immune response [112,113].

On the other hand, dietary treatment with cow’s milk fermented with *Lactobacillus paracasei* CBA L74 (FM-CBAL74) has been associated with high abundance of predicted genes involved in butyrate synthesis in pediatric GI and respiratory infections [114]. Nowadays, as for the use of the genetically modified lactic acid bacteria such as *Lactococcus lactis* and certain species of Lactobacilli, it is possible to create alive recombinant vectors for the progress of new nontoxic mucosal vaccines [115,116].

Personalized probiotic therapy aims to manipulate the GM of the host in order to ameliorate metabolic diseases, asthma, arthritis, and cancer [117,118,119,120].

Several plant polysaccharide complexes present in foods, as previously described, cannot be digested by human enzymes due to their insolubility or to a deficiency of human-encoded hydrolytic enzymes—these food substrates are called prebiotics [121]. Prebiotics are defined as dietary supplements and are resistant starch, β-glucans, inulin, pectin, and other GOS and FOS, and are taken in order to increase the level of beneficial bacteria (i.e., Bifidobacteria and LAB). The prebiotics are compounds that selectively exercise an influence on the GM composition or function, exerting a beneficial effect to the host after bacterial metabolism [122]. However, some bacteria acting as probiotics can degrade them, and the consumption of polysaccharides is important for their growth [109].

However, Khalesi et al. [123] collected the literature on the effectiveness of probiotics in healthy adults, evidencing the failure of ability of probiotics to cause persistent changes in GM [123].

Furthermore, guidelines of clinical practice have not reviewed the safety and potential adverse events that may be encountered when using probiotics in the treatment of different pediatric diseases [124]. Therefore, the suitability of probiotics administration to provide real benefits in healthy adults needs further investigation.

Omics-based analyses suggest for prebiotics a promising therapeutic role in metabolic syndrome or inflammatory bowel syndrome (IBS) [125,126], even though their action mode and their effects on different microbiome ecosystems require further studies. The dietary supplement response, however, exhibits an inter-individual variation, partially due to the microbiome composition [127,128].

In addition, a more complete dietary approach, namely, “personalized nutrition” (PN) utilizes a large specific population’s metadata (including a rich dataset of microbiome features) and bioinformatic tools, which can allow personalized dietary interventions that are able to modify the microbiome by affecting metabolic homeostasis [129].

The GM may be affected by numerous factors, among which diet may assume a fundamental role. The ability to change the GM composition and activity by tailored dietary interventions is not only attractive but also is an encouragement for future research in disease prevention and wellness management [130].

The advances in human genome sequencing and the field of precision nutrition determines precision lifestyle medicine, which can classify a variety of nutrient metabolisms among subgroups (e.g., ethnicity, health status, lifestyle, cultural preferences, and clinical variables) and inter-individual variability in reactions to dietary interventions [131]. Several studies focused on the co-evolution of humans and their GM to understand to what extent the spread of Western diet and lifestyle have impacted our microbial symbionts and how this has affected human health [46,132].

Personalized strategies to propose optimized nutrition are being consolidated, but more investigations are necessary to improve PN knowledge derived from microbiome studies [133].

Another possibility in GM management is the use of synbiotics, in which prebiotics and probiotics are present in synergistic combinations [134,135].

Previous studies have highlighted the fact that synbiotics have a potential heavy effect on GM metabolic activity modulation, more than probiotics or prebiotics themselves [134,136,137].

It has also been reported that synbiotic administration can improve (i) metabolic status (total cholesterol, low density lipoprotein cholesterol (LDL-c), high density lipoprotein cholesterol (HDL-c), triglycerides etc.) [125], and (ii) serum level of liver function enzymes [123] and inflammatory biomarkers [138] by changing the composition and/or function of the GM.

The non-viable bacterial products or metabolic by-products derived from probiotics are called “postbiotics”. Postbiotics are functional products derived from fermentation (i.e., SCFAs, secreted polysaccharides, extracellular polysaccharides (EPS), microbial fractions, functional proteins, cell lysates, teichoic acid, peptidoglycan-derived muropeptides, ethanol, diacetyl, acetaldehydes, and hydrogen peroxide), which could be used in combination with nutritional components to promote health [139]. These metabolic products have a wide range of inhibitory properties towards pathogenic microbes and, therefore, can be used as a substitute of antibiotics [140].

Two common types of postbiotics are represented by paraprobiotics and fermented infant formulas (FIFs). Paraprobiotics, also called ghost probiotics, are non-viable probiotics or inactivated probiotics, generally defined as “inactivated or non-viable microbial cells, when administered in sufficient amounts give benefits to the host” [141]. FIFs are infant formulas fermented by lactic acid-producing or other bacteria, and in many cases do not contain viable bacteria [142].

Therefore, the employment of postbiotic molecules has become a possible strategy for treating many inflammatory diseases through comprehension of microbiota–host metabolism mechanisms. Indeed, these molecules mime the beneficial and therapeutic effect of probiotics, eluding the living microorganisms’ administration to a host with a compromised immune system [109,143].

A potential efficient microbiome intervention is represented by FMT, in which a healthy donor microbiome is transplanted into a patient to correct the individual’s own disease-associated microbiome.

The GM modulation by FMT principally follows the probiotic principle, but the patient will not be treated with specific strains, but instead with a fecal suspension infusion from a healthy donor [144]. Currently, this treatment is approved for recurrent *Clostridium difficile* infections (rCDI) in adulthood, and is now also tested in different clinical procedures for treating numerous pathological conditions, ranging from metabolic and neoplastic to autoimmune disorders [145,146], both in adulthood and childhood. However, transplanting an entire community of microbes involves risks, as the pathogen/pathobiont transmission can generate undesirable effects for the transplanted microbiome [147] and incomplete long-term stabilization of an extraneous microbial configuration when introduced into a new host with a unique genetic, immune, metabolic, and nutritional environment [129]. Further studies are necessary to optimize and improve this technique and to increase FMT usage as clinical treatment beyond rCDI. Clinical trials are necessary to standardize adverse events’ registration, patients’ registers, experimental methods to identify metabolites, and metagenomes associated with different disease treatments by FMT. This is the reason why international consensus and/or recommendation are needed for donor screening for a wide range of diseases [127,128,129]. Recently, to support the importance of donor screening, it has been suggested that the FMT success depend on the microbial diversity and microbial composition of the donor, generating the hypothesis of the existence of FMT super-donors. The determination and characterization of super-donor gut microbes will help to understand bacterial components of diseases and allow for the creation of more targeted approaches in the future [148,149,150,151].

## 5. Conclusions and Perspective

The research thus far reveals, summarizing the previous results, that the GM can respond to altered diet, potentially facilitating the diversity of human dietary lifestyles.

Diet is one of the most important driving forces able to shape the GM. Dietary interventions and targeted nutritional therapies, such as medical foods, dietary supplements, living microorganisms, nutraceutical food, and FMT, could provide a great promise for the prevention and treatment of microbiota-related diseases. However, much experimental research is needed before these opportunities can be fully realized. Effects of specific nutrients need to be assessed in clinical trials. Finally, the microbiomics field has undergone a massive revolution by the identification of variables that lead to the development of bacterial community structure and functionality, as well as in terms of understanding how these gut microbial ecosystems and their metabolism of dietary ingredients can influence both healthy human status and disorders. It will in future be possible to define new types of therapies that could be developed with the focus on creating “personalized” diets and microbiomes in order to promote health.
